# Scorpion Venom Polypeptide Inhibits Pulmonary Epithelial-Mesenchymal Transition in Systemic Sclerosis-Interstitial Lung Disease Model Mice by Intervening TGF-*β*1/Smad Signaling Pathway

**DOI:** 10.1155/2022/6557486

**Published:** 2022-04-13

**Authors:** Yan Zhang, Liping Xu, Qiang Chen, Tianrong Guan, Na Lin, Danyang Xu, Lihong Lu, Qiaoding Dai, Xinwei Song

**Affiliations:** ^1^Department of Rheumatism and Immunology, The First Affiliated Hospital of Zhejiang Chinese Medical University/Zhejiang Provincial Hospital of Traditional Chinese Medicine, No. 54 Youdian Road Shangcheng District, Hangzhou, Zhejiang 310006, China; ^2^Department of Orthopedics, Affiliated Xiaoshan Hospital, Hangzhou Normal University, No. 728 Yucai North Road Xiaoshan District, Hangzhou, Zhejiang 311200, China

## Abstract

**Objective:**

Interstitial lung disease (ILD) is an important complication of systemic sclerosis (SSc). The aim of this study was to investigate the effect and possible mechanism of polypeptide extract of scorpion venom (PESV) on SSc-ILD.

**Methods:**

C57/BL6 mice were injected with bleomycin to establish a SSc-ILD model. Different concentrations of PESV solution were administered to SSc-ILD mice, and dexamethasone was used as a positive control. H&E staining and Masson staining were used to observe the pathological changes. The TGF-*β*1 expression level was detected by immunohistochemistry. The expression of epithelial-mesenchymal transition (EMT)-related proteins was detected by Western blot, and the expression of TGF-*β*1/Smad pathway-related proteins was also detected. The content of inflammatory cytokines in serum and BALF was determined by ELISA.

**Results:**

Pathological analysis showed that PESV could alleviate SSc-ILD-induced pulmonary inflammation and fibrosis. Compared with the model group, the content of inflammatory cytokines IL-6 and TNF-*α* significantly decreased after PESV treatment. PESV could increase the expression of epithelial marker (E-cadherin) and reduce the expression of interstitial markers (collagen I, vimentin, N-cadherin, and a-SMA). In addition, PESV could reduce the expression level of TGF-*β*1/Smad pathway-related protein.

**Conclusion:**

PESV can attenuate SSc-ILD by regulating EMT, and the effect was linked to the TGF-*β*1/Smad signaling pathway, which indicated that PESV may serve as a candidate drug for SSc-ILD.

## 1. Introduction

Systemic sclerosis (SSc) is an immune-mediated rheumatic disease characterized by fibrosis of the skin and internal organs and vasculopathy [1]. Despite evidence of improved survival, SSc still has a higher mortality than any other rheumatic diseases [2]. Up to 80% of SSc patients have interstitial lung disease (ILD) on high-resolution computed tomography (HRCT) scan, and 30–40% of SSc patients have clinically significant ILD; SSc-associated ILD is the leading cause of disease-related mortality [3]. The basic pathological changes in ILD are interstitial fibrosis, diffuse parenchymal, and alveolar inflammation. At present, there is no effective drug for the treatment of SSc-ILD [2]. The pathogenesis of SSc-ILD has not been completely unraveled, but there is a wide belief that epithelial-mesenchymal transition (EMT) may take place in the skin of SSc patients and play a role in the pathogenesis of ILD [4, 5]. Hence, inhibition of EMT may serve as a rational strategy to treat SSc-ILD.

TGF-*β*1 is a profibrotic factor, and the TGF-*β*1/Smad signaling pathway involved is also closely related to fibrosis and EMT [6]. Qian et al. have demonstrated that the TGF-*β* signaling pathway inhibitor can obviously ameliorate pulmonary fibrosis in bleomycin-induced pulmonary fibrosis mice [7]. Guan et al. also reported that ginsenoside Rg1 alleviates cigarette smoke-induced pulmonary EMT by suppressing the TGF-*β*1/Smad pathway both in vivo and in vitro [8]. In addition, studies suggested that the TGF-*β*1/Smad pathway may regulate airway EMT and physiological obstruction through fibrosis [9]. Hence, inhibition of the TGF-*β*1/Smad signaling pathway may help to prevent SSc-ILD.

Scorpion, a kind of precious traditional Chinese medicine (TCM), has the effect of antitumor, sedative, and analgesic, as well as anticonvulsant, which is commonly used in the treatment of diseases such as malignant tumors, convulsions, and rheumatism [10]. The chemical constituents of scorpion venom mainly contain the polypeptide extract of scorpion venom (PESV), water-soluble components, and fat-soluble components [11]. The chemistry structural diversity of PESV contributes to greater functions, including anti-inflammatory, antibacterial, and antifibrotic effects [12]. Previous studies have found that PESV is able to reduce the expression of TGF-*β*1 [13]. PESV can induce the apoptosis of the tumor cells by inhibiting the expression of VEGF and TGF-*β*1 [14]. However, the specific function and detailed mechanism of PESV in SSc-ILD are poorly understood [15].

Hence, in this study, we hypothesize that PESV could improve pulmonary EMT by regulating the TGF-*β*1/Smad signal pathway, thus to alleviate SSc-ILD. We established a mouse model of SSc-ILD and intervened with different concentrations of PESV, with the aim to study the specific effect of PESV on the symptoms of SSc-ILD and its detailed mechanism.

## 2. Materials and Methods

### 2.1. Grouping and Establishment of Experimental Animals

Forty-eight healthy adult female SPF grade C57/BL6 mice aged 6–8 weeks with the bodyweight of 20–22 g were randomly divided into six groups: the normal control group (NC group), bleomycin-induced SSc-ILD model group (BLM group), low-dose PESV intervention group (PESV-L group), medium-dose PESV intervention group (PESV-M group), high-dose PESV intervention group (PESV-H group), and dexamethasone intervention group (DXM group), with eight mice in each group. This animal experimental program has been reviewed by the Experimental Animal Management and Ethics Committee of Zhejiang University of Traditional Chinese Medicine and conforms to the principles of animal protection, animal welfare, and ethics, as well as the relevant provisions of China's National Experimental Animal Welfare Ethics (Animal Experiment Ethics Approval Number: IACUC-20210524-03).

SSc-ILD animal modeling in this study was based on the reports of Aso Y [16] and Yamamoto T. et al [17]. After removing 2.0 × 2.0 cm^2^ hair from the center of the back in each mouse with 10% sodium sulfide solution, mice in the NC group were subcutaneously injected with 0.1 ml of normal saline, and the rest of the mice were subcutaneously injected with 0.1 ml of bleomycin solution (1 mg/ml, Hanhui Pharmaceutical Co., Ltd., 2006741) once a day for 4 weeks to establish the mouse SSc-ILD model. In addition, the mice in the PESV-L group, PESV-M group, PESV-H group, and DXM group were intragastrically administered with 5 mg/kg PESV solution (Zhengzhou Licheng Biotechnology Co., Ltd.), 10 mg/kg PESV solution, 20 mg/kg PESV solution, and 0.15 mg/kg dexamethasone sodium phosphate injection (Suicheng Pharmaceutical Solution Co., Ltd.) for 4 weeks from the day of modeling, respectively [18]. The mental state, activity, respiration, fur change, food intake, water intake, and other general conditions of mice in each group were observed during the experiment.

### 2.2. Bronchoalveolar Lavage Fluid (BALF), Lung, and Serum Collection

After 4 weeks of administration, the mice in each group were sacrificed, and their blood samples were collected by the eyeball removal method and placed in sterilized EP tubes overnight (4°C). After coagulation, the blood was centrifuged (4°C, 10 min, 2000 rpm). The supernatant was collected and stored at −80°C.

The skin on the anterior cervical region of the mice was incised. A red intravenous catheter was placed at the distal end of the trachea and ligated and fixed. One milliliter of normal saline was then infused into the lung tissue. After the lung was significantly filled, hold for 10 s to pump back, which was repeated three times. Finally, the collected BALF was centrifuged (4°C, 800 rpm, 5 min) and stored at −80°C until use. After that, lung tissues from the mice were dissected, a portion was fixed in 10% neutral formaldehyde (Fuzhou Feijing Biotechnology Co., Ltd.), and another portion was stored at −80°C.

### 2.3. H&E Staining

Lung tissue samples of mice fixed in formaldehyde were cut into 4 *μ*m thick paraffin sections. Subsequently, the sections were dewaxed by xylene and rehydrated with ethanol. Hematoxylin staining solution was added, followed by differentiation (0.5% hydrochloric acid in alcohol, 10 s) and reversion to blue (1% diluted ammonia, 90 s). oEsin staining solution (60 s) was then added for staining, and the results were observed under a microscope after fixation with neutral resin.

### 2.4. Masson's Staining

The lung tissue of mice in each group was deparaffinized to water after sectioning, stained with iron hematoxylin staining solution (10 min), refluxed with blue solution (5 min), and finally stained with red staining solution (15 min). After differentiation (1% acetic acid, 10 s), the sections were fixed with neutral gum and observed under a microscope.

### 2.5. Immunohistochemical Staining

Lung tissue sections from mice in each group were deparaffinized to water and subjected to antigen heat retrieval. The primary TGF-*β*1 antibody (ImmunoWay Biotechnology Company, USA) was added followed by overnight incubation at 4°C. Secondary antibodies were added the following day and incubated for half an hour at 37°C. Then, DAB chromogenic solution was added, and the staining results were observed microscopically at 200x field after counterstaining with hematoxylin.

### 2.6. Western Blot

The lung tissue samples of mice in each group were crushed, lysed, and centrifuged. The total protein concentration was measured with a BCA kit, followed by SDS-PAGE electrophoresis, and then transferred to a PVDF membrane. At the end of the transfer membrane, blocking was performed at room temperature for 1 h with 5% nonfat dry milk, and then, the membrane was incubated at 4°C overnight with corresponding primary antibodies (E-cadherin antibody (Affinity, AF0131), collagen I antibody (Affinity, AF7001), vimentin antibody (Affinity, AF7013), N-cadherin antibody (Affinity, AF4039), *a*-SMA antibody (Affinity, BF9212), TGF-*β*1 antibody (Affinity, AF1027), T*β*RI antibody (Affinity, DF7309), T*β*RII antibody (Affinity, DF13307), phospho-Smad2 (Ser250) antibody (Affinity, AF3450), Smad2 antibody (Affinity, AF6449), phospho-Smad3 (Ser423 + Ser425) antibody (Affinity, AF8315), Smad3 antibody (Affinity, AF6362), and Smad7 antibody (Affinity, AF5147)). After incubation with an HRP-labeled secondary antibody at room temperature for 1 h, the immunoreactive bands were visualized by developing solution. After scanning, the gray value of each band was determined by image analysis software, and the ratio with the internal reference *β*-actin was used as the basis for semiquantitative analysis.

### 2.7. ELISA Test

Take the standby BALF and serum, place them at room temperature for 20 min, and then add distilled water to dilute 20 times the concentrated washing solution into original washing solution. The corresponding inflammatory factor concentrations in BALF were measured according to the manufacturer's instructions of the ELISA kit. The tested ELISA kits include the mouse interleukin-6 (IL-6) ELISA kit (ELK1157, ELK Biotechnology), mouse transforming growth factor-beta 1 (TGF-*β*1) ELISA kit (ELK1186, ELK Biotechnology), and mouse tumor necrosis factor-alpha (TNF-*α*) ELISA kit (ELK1387, ELK Biotechnology).

### 2.8. Statistical Analysis

All data from this experiment were analyzed and processed using SPSS 16.0 and GraphpadPrism7 software. All data were expressed as mean ± standard deviation ($\bar{x}\pm s$), *P* < 0.05 was considered statistically significant. One-way ANOVA (analysis of variance) was used for multiple groups of measurement data, and the SNK test was used for comparison. The Kruskal–Wallis H test was used for unequal or nonnormal variance.

## 3. Results

### 3.1. General Condition of the Mice

Mice in the NC group showed good mental status, normal activity, shiny coat color, soft skin, and normal diet. Mice in the BLM group had symptoms consistent with SSc, including inactivity, apathy, crouching at corners, tarnished coat color, hardened skin epidermis, and reduced eating. While, these symptoms were alleviated to varying degrees in the mice of the drug-treated groups.

### 3.2. Pathological Change of Lung Tissues

**Figure 1:**
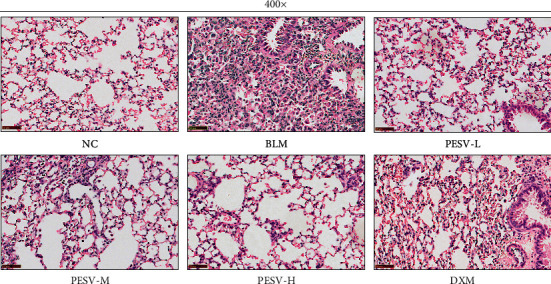
Pathological change of lung tissue in mice of each group (magnification: 400-fold, scale bar = 50 *μ*m). NC, normal control group; BLM, bleomycin-induced systemic sclerosis model group; PESV-L, low-dose PESV intervention group; PESV-M, medium-dose PESV intervention group; PESV-H, high-dose PESV intervention group; DXM, dexamethasone intervention group.

The lung tissue of the mice in the NC group was clear and the alveolar structure was intact. In contrast, the BLM group clearly had a large number of fibrocyte and inflammatory cell infiltration, increased lung macrophages, significantly thicker septa between alveoli, and severely destroyed alveolar structure. The degree of lung tissue lesion in the PESV-L group, PESV-M group, PESV-H group, and DXM group was significantly alleviated compared with the BLM group. The pathological change was improved to different extents after treatment with different concentrations of PESV in a dose-dependent manner (Figure 1).

### 3.3. Fibrotic Changes in Lung Tissue

**Figure 2:**
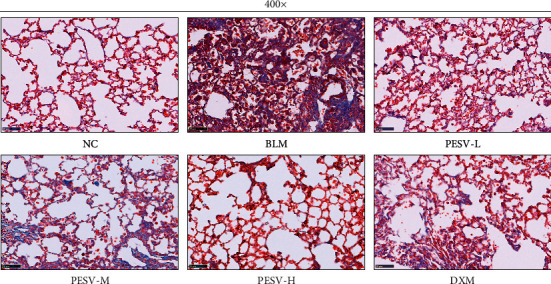
Masson staining of lung tissue from mice in each group (400-fold, scale bar = 50 *μ*m). NC, normal control group; BLM, bleomycin-induced systemic sclerosis model group; PESV-L, low-dose PESV intervention group; PESV-M, medium-dose PESV intervention group; PESV-H, high-dose PESV intervention group; DXM, dexamethasone intervention group.

As shown Figure 2, Masson's staining of lung tissue showed that the alveolar structure of the NC group was normal without significant fibrotic change. However, in the BLM group, the alveolar structure was severely destroyed, and a mass of fibrous hyperplasia stained with blue was observed, showing the collagen bundle formation of the lung. The results of the PESV-L, PESV-M, PESV-H, and DXM groups showed that although fibrous hyperplasia was still observed, it was significantly milder than that of the BLM group.

### 3.4. Content of Inflammatory Cytokines IL-6, TNF-*α*, and TGF-*β*1 in Serum and BALF

**Figure 3:**
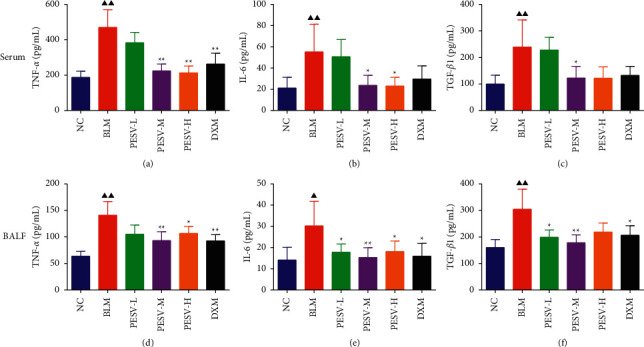
Content of inflammatory cytokines TNF-*α*, IL-6, and TGF-*β*1 in serum ((a), (b), (c)) and BALF ((d), (e), (f)) of mice in each group. NC, normal control group; BLM, bleomycin-induced systemic sclerosis model group; PESV-L, low-dose PESV intervention group; PESV-M, medium-dose PESV intervention group; PESV-H, high-dose PESV intervention group; DXM, dexamethasone intervention group. Compared with the NC group, ^▲^*P* < 0.05, ^▲▲^*P* < 0.01; compared with the BLM group, ^★^*P* < 0.05, ^★★^*P* < 0.01. Data represent the mean ± SEM, *n* = 3.

The content of IL-6, TNF-*α*, and TGF-*β*1 in serum and BALF was detected by ELISA. Results are shown in Figure 3. It can be seen that the change trend for the content of these inflammatory cytokines in serum and BALF was coincident. Compared with the NC group, the content of inflammatory factors TGF-*β*1, IL-6, and TNF-*α* in serum and BALF was significantly increased in the BLM group (*P* < 0.05 or *P* < 0.01). Compared with the BLM group, the content of these factors was significantly decreased after treating with PESV and positive drug (dexamethasone) (*P* < 0.05 or *P* < 0.01). The content of inflammatory factors was reduced to different extents after treating with different concentrations of PESV in a dose-dependent manner.

### 3.5. Expression of EMT-Related Proteins in Lung Tissue

**Figure 4:**
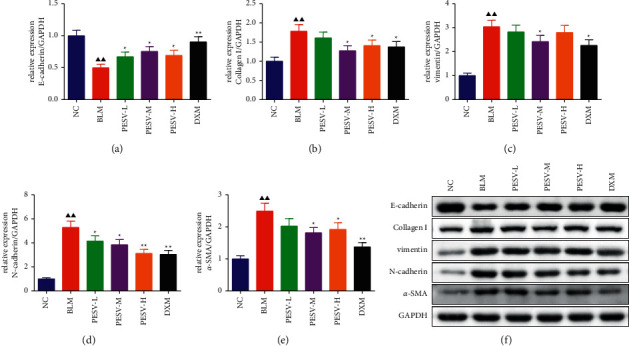
The expression levels of E-cadherin (a), collagen I (b), vimentin (c), N-cadherin (d), and *a*-SMA (e) protein and their protein band (f) in the lung tissue of mice in each group. NC, normal control group; BLM, bleomycin-induced systemic sclerosis model group; PESV-L, low-dose PESV intervention group; PESV-M, medium-dose PESV intervention group; PESV-H, high-dose PESV intervention group; DXM, dexamethasone intervention group. Compared with the NC group, ^▲^*P* < 0.05, ^▲▲^*P* < 0.01; compared with the BLM group, ^★^*P* < 0.05, ^★★^*P* < 0.01. Data represent the mean ± SEM, *n* = 3.

The results of Western blot showed that the protein expression of an epithelial marker (E-cadherin) in the lung tissue of mice in the BLM group was significantly lower than that in the NC group (*P* < 0.01), while the protein expression levels of mesenchymal markers (collagen I, vimentin, N-cadherin, and *a*-SMA) were significantly higher than those in the NC group (*P* < 0.01). After treatment with different concentrations of PESV and DXM, the situations were reversed (Figure 4).

### 3.6. Expression of TGF-*β*1 in Lung Tissue

**Figure 5:**
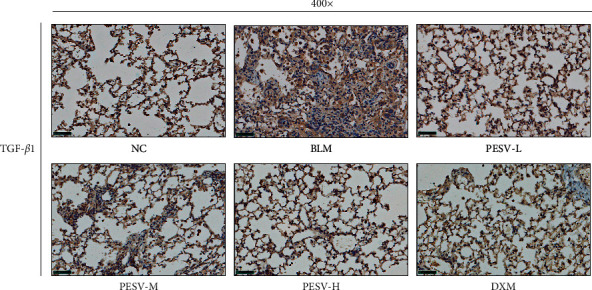
Immunohistochemical staining of TGF-*β*1 in the lung tissue of mice in each group (400-fold, scale bar = 50 *μ*m). NC, normal control group; BLM, bleomycin-induced systemic sclerosis model group; PESV-L, low-dose PESV intervention group; PESV-M, medium-dose PESV intervention group; PESV-H, high-dose PESV intervention group; DXM, dexamethasone intervention group.

It can be seen from Figure 5 that the expression level of TGF-*β*1 protein in the BLM group was significantly higher than that in the NC group, with obviously more brown stained cells. While, the expression levels of TGF-*β*1 protein in the PESV-L group, PESV-M group, PESV-H group, and DXM groups were significantly lower than that in the BLM group.

### 3.7. Expression of TGF-*β*1/Smad Signaling Pathway-Related Proteins in Lung Tissue

**Figure 6:**
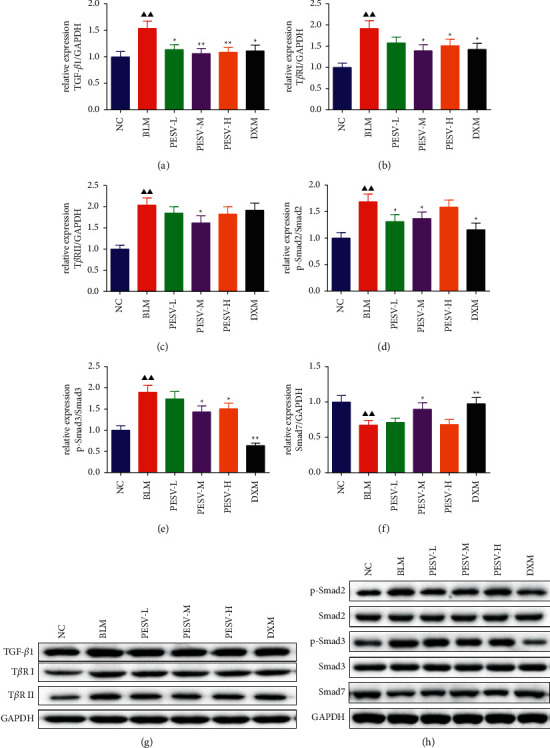
The expression levels of TGF-*β*1 (a), T*β*RI (b), T*β*RII (c), p-Smad2 (d), p-Smad3 (e), and Smad7 (f) protein and their protein band ((g), (h)) in the lung tissue of mice in each group. NC, normal control group; BLM, bleomycin-induced systemic sclerosis model group; PESV-L, low-dose PESV intervention group; PESV-M, medium-dose PESV intervention group; PESV-H, high-dose PESV intervention group; DXM, dexamethasone intervention group. Compared with the NC group, ^▲^*P* < 0.05, ^▲▲^*P* < 0.01; compared with the BLM group, ^★^*P* < 0.05, ^★★^*P* < 0.01. Data represent the mean ± SEM, *n* = 6.

As shown in Figure 6, the expression levels of TGF-*β*1, T*β*RI, T*β*RII, p-Smad2, and p-Smad3 protein in the lung tissue of the BLM group mice were significantly higher than those in the NC group (*P* < 0.01), and the expression level of Smad7 protein was significantly lower (*P* < 0.01). However, after treating with different concentrations of PESV and DXM, the results were reversed, compared with the BLM group.

## 4. Discussion

SSc is a rheumatic immune disease characterized by skin and organ fibrosis [19]. Studies have concluded that the pathogenesis of SSc (including skin, lung, and other tissue fibrosis) is associated with damage of endothelial cells and activation of collagen, which stimulates fibroblast synthesis and then leads to fibrosis of blood vessel walls and tissues [20]. The key mechanism of SSc-ILD pathogenesis is persistent lung damage caused by alveoli macrophage activation, which induces the release of a variety of profibrosis cytokines, prompts the conversion of pulmonary epithelial cells to interstitial cells (EMT), and eventually results in the formation of pulmonary fibrosis [21]. Recently, TCM has been more and more accepted in the treatment of pulmonary fibrosis worldwide [22]; Yangyin Yiqi Mixture [23], *Nervilia fordii* extract [24], and Inulae Flos [25] have a good effect on pulmonary fibrosis with few side effects. Although the active role of PESV on SSc-ILD is unclear, a published study has proved that PESV obviously improves fibrosis in rats with doxorubicin-induced acute cardiotoxicity [26]. In addition, Xu et al. also demonstrated that PESV is effective in recovering immunosurveillance and intervening immune escape of lung cancer by decreasing the level of VEGF (a marker of endothelial dysfunction), TGF-*β*1 (profibrotic factor), and IL-10 [27].

Recently, various cytokine level abnormalities have been found in serum and affected lung tissue in SSc-ILD patients, such as TGF-*β*1, TNF-*α*, and IL-6, and these cytokines are thought to promote fibroblast activation and EMT, which plays a key role in the occurrence of SSc-ILD inflammation and fibrosis [28]. At present, TGF-*β*1 is the most in-depth and important cytokine in the cytokine network involved in SSc-ILD, which regulates the expression of matrix metalloproteinases (MMP) and extracellular matrix (ECM) by activating EMT-related transcription factors to promote epithelial cell apoptosis, migration, and conversion to mesenchymal cells [29, 30]. Furthermore, IL-6 and TNF-*α* are strongly related to the development of lung fibrosis and EMT. Specifically, the effect of IL-6 on EMT is mediated by activation of STAT365, and TNF-*α* can initiate EMT by upregulating and stabilizing Snail and Twist [31]. It has been shown that PESV, an active ingredient of *Buthus martensii* Karsch, can effectively improve the expression of E-cadherin in leukemia mice and can interfere with the growth and metastasis of Lewis lung carcinomas in mice by inhibiting MMP-9 [32, 33]. PESV also has a significant inhibitory effect on the EMT process of liver cancer cells. It can reduce the invasiveness of liver cancer cells and inhibit further metastasis of liver cancer [34]. The above studies suggest that PESV may have the effect of inhibiting EMT and reducing tissue fibrosis.

In our study, the SSc-ILD model of mice was established by subcutaneous injection of bleomycin. The results showed the skin in the back injection area of mice in the BLM group was hardening, and the results of H&E and Masson's staining of pulmonary tissues showed alveoli inflammation and fibrosis, respectively, which were consistent with the results of Wolflin L et al. [35], indicating the success of SSc-ILD model construction. Our further results suggested that PESV could significantly improve the typical symptoms and pathological changes in the lung of SSc-ILD model mice. The content of TGF-*β*1, TNF-*α*, and IL-6 in serum and BALF was detected by the ELISA method, and the results showed that compared with the NC group, the content of inflammatory factors TGF-*β*1, TNF-*α*, and IL-6 in the model group was significantly increased. However, after treatment with different concentrations of PESV, the content of the abovementioned inflammatory factors was decreased in a dose-dependent manner, suggesting that PESV played a vital role in improving inflammation of the whole body and lung of SSc-ILD model mice. In addition, our study found that PESV had an active effect on epithelial cell marker E-cadherin and inhibited mesenchymal cell markers collagen I, vimentin, N-cadherin, and *a*-SMA, suggesting that PESV may inhibit the EMT process in SSc-ILD model mice.

TGF-*β*1 is one of the common factors that triggers pulmonary fibrosis, and TGF-*β*1 overexpression causes pulmonary fibrosis, while TGF-*β*1 inhibitors can reduce the degree of pulmonary fibrosis [6]. Numerous studies have confirmed that TGF-*β*1 can promote tissue fibrosis by inducing EMT [36]. EMT is a critically important event in tissue fibrosis; it is characterized by the inactivation of the epithelial cell markers, including E-cadherin, ZO-1, and the activation of mesenchymal cell markers, such as collagen I, N-cadherin, vimentin, *a*-SMA, and fibronectin [37]. Furthermore, we detected significantly a higher TGF-*β*1 protein expression in the BLM group than in NC and PESV intervention groups by examining the expression of TGF-*β*1 and EMT-related protein in mice of each group. The expression of epithelial cell marker E-cadherin in the lung tissue of mice in the BLM group was significantly lower than that of the NC and PESV groups. The mesenchymal cell marker collagen I, vimentin, N-cadherin, and *a*-SMA protein expression were significantly higher than those in the NC and PESV groups. It was shown that TGF-*β*1 expression was positively correlated with the EMT process, and it was further shown that PESV could inhibit TGF-*β*1 expression and EMT process.

Additionally, the induction of EMT by TGF-*β*1 is closely linked with Smad2/3 and Smad7 [38]. Lee SH et al. reported that TGF-*β*1 inhibits fibrosis by inducing the phosphorylation of Smad2/3 [39]. Lee CM et al. proved that the mediating effect of TGF-*β*1 on the fibrotic response is strongly associated with the inhibition of Smad7 expression [40]. We experimentally found that the expression of TGF-*β*1/Smad2/3 pathway-related proteins in SSc-ILD model mice was significantly higher than that in the control group. Nevertheless, the expression of pathway-related proteins was obviously after PESV treatment, which indicated that the inhibitory effect of PESV on EMT in SSc-ILD model mice may be related to the TGF-*β*1/Smad pathway.

In previous studies on PESV, there were few mechanisms related to the TGF-*β*1 pathway, but the regulation of PESV on TGF-*β*1 has been reflected in a few literature [27]. In this study, we found that PESV has a significant inhibitory effect on the expression of TGF-*β*1 and its receptor. Combined with the promoting effect of TGF-*β*1 on EMT in SSc-ILD model mice, it is indicated that PESV may achieve the inhibition of EMT by regulating the TGF-*β*1 pathway, thereby alleviating pulmonary inflammation and interstitial fibrosis in mice.

## 5. Conclusions

In conclusion, this study investigated the therapeutic potential of PESV for SSc-ILD. Specifically, we demonstrated that PESV could inhibit SSc-ILD-induced EMT and alleviate pulmonary inflammation and interstitial fibrosis in model mice by the intervention of the TGF-*β*1/Smad signaling pathway. These results suggest that PESV may be used as a potential drug for the treatment of SSc-ILD. However, further in-depth studies are needed to confirm the clear role of PESV in SSc-ILD.

## Data Availability

The data used to support the findings of this study are included within the article.
